# The Plasmid pEX18Gm Indirectly Increases *Caenorhabditis elegans* Fecundity by Accelerating Bacterial Methionine Synthesis

**DOI:** 10.3390/ijms23095003

**Published:** 2022-04-30

**Authors:** Rui Guo, Gen Li, Leilei Lu, Shan Sun, Ting Liu, Mengsha Li, Yong Zheng, Albertha J. M. Walhout, Jun Wu, Huixin Li

**Affiliations:** 1 College of Resources and Environmental Sciences, Nanjing Agricultural University, Nanjing 210095, China; 2015203007@njau.edu.cn (R.G.); 2016203005@njau.edu.cn (G.L.); 2019203038@njau.edu.cn (L.L.); 2019203039@njau.edu.cn (S.S.); ting.liu@njau.edu.cn (T.L.); 2 Department of Systems Biology, University of Massachusetts Chan Medical School, Worcester, MA 01605, USA; marian.walhout@umassmed.edu; 3 College of Science & Technology, Ningbo University, Cixi 315300, China; limonms@163.com; 4 Key Laboratory of Aquatic Botany and Watershed Ecology, Wuhan Botanical Garden, Chinese Academy of Sciences, Wuhan 430074, China; zhengyong@wbgcas.cn; 5 Jiangsu Collaborative Innovation Center for Solid Organic Waste Resource Utilization, Nanjing 210014, China

**Keywords:** plasmid–host interaction, bacteria, *C. elegans*, fecundity, multi-species, RNA-seq, methionine, HPLC-MS/MS

## Abstract

Plasmids are mostly found in bacteria as extrachromosomal genetic elements and are widely used in genetic engineering. Exploring the mechanisms of plasmid–host interaction can provide crucial information for the application of plasmids in genetic engineering. However, many studies have generally focused on the influence of plasmids on their bacterial hosts, and the effects of plasmids on bacteria-feeding animals have not been explored in detail. Here, we use a “plasmid–bacteria–*Caenorhabditis elegans*” model to explore the impact of plasmids on their host bacteria and bacterivorous nematodes. First, the phenotypic responses of *C. elegans* were observed by feeding *Escherichia coli* OP50 harboring different types of plasmids. We found that *E. coli* OP50 harboring plasmid pEX18Gm unexpectedly increases the fecundity of *C. elegans*. Subsequently, we found that the plasmid pEX18Gm indirectly affects *C. elegans* fecundity via bacterial metabolism. To explore the underlying regulatory mechanism, we performed bacterial RNA sequencing and performed in-depth analysis. We demonstrated that the plasmid pEX18Gm upregulates the transcription of methionine synthase gene *metH* in the bacteria, which results in an increase in methionine that supports *C. elegans* fecundity. Additionally, we found that a pEX18Gm-induced increase in *C. elegans* can occur in different bacterial species. Our findings highlight the plasmid–bacteria–*C. elegans* model to reveal the mechanism of plasmids’ effects on their host and provide a new pattern for systematically studying the interaction between plasmids and multi-species.

## 1. Introduction

Plasmids are genetic particles that replicate autonomously outside the chromosome and are found in many organisms, most notably bacteria [1]. Due to the properties of plasmids, they are widely used in genetic engineering to confer a new genotypic and phenotypic trait on their hosts [2,3,4,5]. Many studies on plasmid–host interactions provide a guarantee for the rational application of plasmids, and it helps to understand the ecological functions of plasmids. Plasmids usually entail a metabolic burden that reduces the reproductive rate of its host bacteria [6,7]. For instance, the plasmid pDM reduces the activities of glucose-6-phosphate dehydrogenase, fructose 1,6-diphosphate aldolase, and fructose 1,6-diphosphatase and decreases the growth rate of *Escherichia coli* DH5α [8]. Moreover, plasmids can improve the survival and tolerance of the host. For example, F and certain R plasmids have been shown to decrease the rate of DNA synthesis and result in the survival increase in nitrogen mustard-treated F- cells [9,10]. The plasmid ColIB enhances the resistance of *Salmonella typhimurium* to the lethal action of UV light and increases the UV-induced mutability level [11,12]. Additionally, plasmids can alter the expression of chromosomal genes involved in a wide variety of bacterial cellular functions including metabolism, respiration, secretion systems, signaling, translation, transcription, motility, the tricarboxylic acid (TCA) cycle and iron acquisition [13]. Currently, the rapid development of omics technologies provides important advantages for analyzing the effects of plasmids on host physiology. Extensive transcriptome data indicate that plasmids can affect the expression of the chromosomal genes and the levels of cell metabolism [14,15,16]. Microarray experiments show that approximately 4% of gene expression in *E. coli* MG1655 is affected by the presence of the F-plasmid, including the methionine and leucine metabolic pathways [17]. The results of the combined transcriptome and metabolome analysis show that diverse plasmids alter the expression of a common set of metabolic genes in *Pseudomonas aeruginosa* PAO1 and produce convergent changes in host cell metabolism [18]. However, these studies generally focused on the influence of plasmids on their bacterial hosts, and the effects of plasmids on bacteria-feeding animals have not been explored in detail.

*Caenorhabditis elegans* and its bacterial diet provide a facile system for studying interspecies interactions [19,20]. This is all due to the fact that *C. elegans* can thrive and reproduce on a single bacterial diet, its short life history, and its phenotype that is extremely easy to monitor under a microscope [21]. Bacterial metabolic changes can affect *C. elegans* life history traits such as lifespan, fecundity and developmental rate. For instance, feeding nitric oxide-deficient *B. subtilis* shortens *C. elegans* lifespan [22]. Feeding *E. coli* mutants in cytochrome *bo* oxidase complex to *C. elegans* causes developmental delay [23]. Furthermore, *C. elegans* are sensitive and can respond to minor dietary changes. For example, supplementation of vitamin B12 to *E.coli* OP50, which harbors low levels of this cofactor, accelerates *C. elegans* development [24]. Feeding *E. coli* HT115 Δ*aroD* mutants with low folate levels extends the *C. elegans* lifespan [25]. Therefore, *C. elegans* is an ideal agent organism for exploring the effects of plasmids on multi-species.

Here, we use *C. elegans* and its bacterial diet with different plasmids (defined as “plasmid–bacteria–*C. elegans*”) as a model to explore the impact of plasmids on the physiology of both the bacteria and the animal. We surprisingly found that *C. elegans* fed *E. coli* OP50 harboring plasmid pEX18Gm produce more offspring than those fed *E. coli* OP50 without any plasmids. Furthermore, we found that the plasmid pEX18Gm affects the methionine synthesis in bacteria, resulting in an increase in methionine availability in *C. elegans* and a resultant increase in its fecundity. Our finding provides a striking example and proposes a new approach to study plasmid interactions with multi-species.

## 2. Results

### 2.1. E. coli OP50 Harboring pEX18Gm Increases C. elegans Fecundity

**Figure 1:**
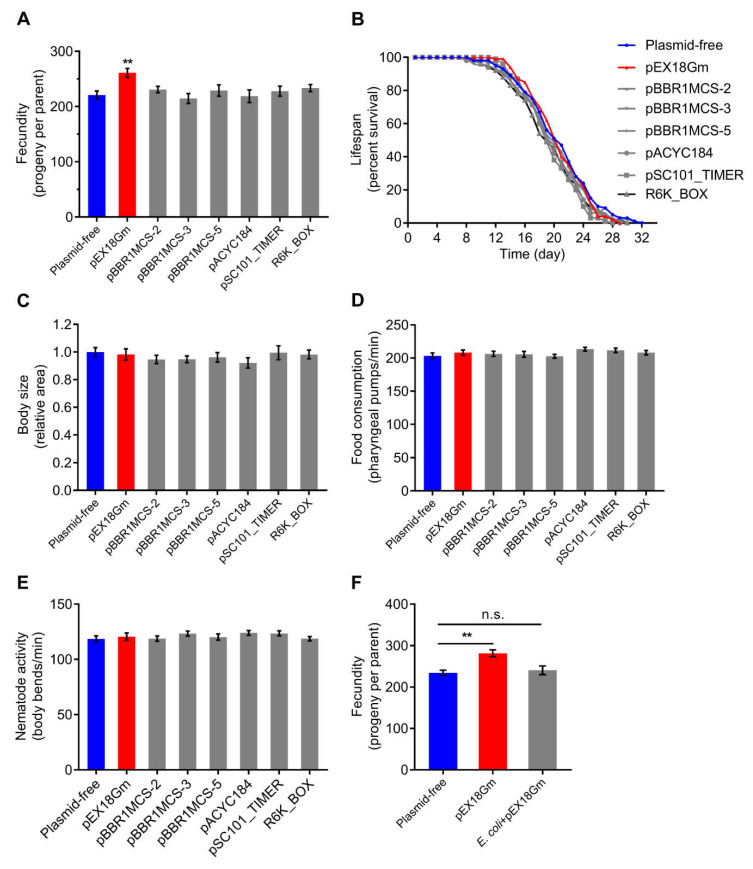
The response of *C. elegans* life-history traits to plasmids. Plasmid-free means *E. coli* OP50 does not harbor any plasmid. Different plasmids are indicated on the x axis. Plasmids were introduced into *E. coli* OP50 as a diet to feed *C. elegans*. (**A**) Fecundity of *C. elegans* on different diets. (**B**) Lifespan of adult animals fed different diets. (**C**) Relative body size of *C. elegans* on different diets. Animal body size was measured by ImageJ and was normalized to the body size of animals grown on the diet of plasmid-free *E. coli* OP50. (**D**) Food consumption of *C. elegans* on different diets. Food intake as measured by pharyngeal pumping rate. (**E**) Nematode activity of *C. elegans* on different diets. Animals’ activity as measured by body bending rate. (**F**) Fecundity of *C. elegans* directly fed on pEX18Gm plasmid. *E. coli* + pEX18Gm indicates the mixed diet that mixes *E. coli* OP50 with pEX18Gm plasmid in vitro (plasmid was extracted from an equal volume of *E. coli* OP50 harboring pEX18Gm). Error bars indicate ± standard error of the mean (SEM). ** *p* < 0.01 by Tukey’s multiple comparison test; n.s. indicates not significant.

First, we collected seven plasmids (pEX18Gm, pBBR1MCS-2, pBBR1MCS-3, pBBR1MCS-5, pACYC184, pSC101_TIMER, and R6K_BOX), involving different sizes, different antibiotic resistances, different replicons, and different plasmid copy numbers (Table 1). To test the response of *C. elegans* phenotype to plasmids, we fed *C. elegans* with *E. coli* OP50 harboring different types of plasmids, and measured life history traits. We found that, relative to animals fed bacteria without any plasmid, animals fed *E. coli* OP50 harboring the pEX18Gm increased *C. elegans* fecundity by nearly 20% without affecting lifespan, body size, food consumption or nematode activity (Figure 1A–E). No effect on *C. elegans* phenotype was found for the other plasmids (Figure 1A–E). Importantly, the presence of pEX18Gm had no significant effect on the growth rate of *E. coli* OP50 (Figure S1). This indicates that it is not the amount of food that confers the change in fecundity. Further, we mixed plasmid-free *E. coli* OP50 with pEX18Gm in vitro and found that this mixture did not increase *C. elegans* fecundity (Figure 1F). The combined findings suggest that pEX18Gm affects *C. elegans* fecundity via changing the metabolism of bacteria.

### 2.2. RNA-seq Analysis of E. coli Strains

**Figure 2:**
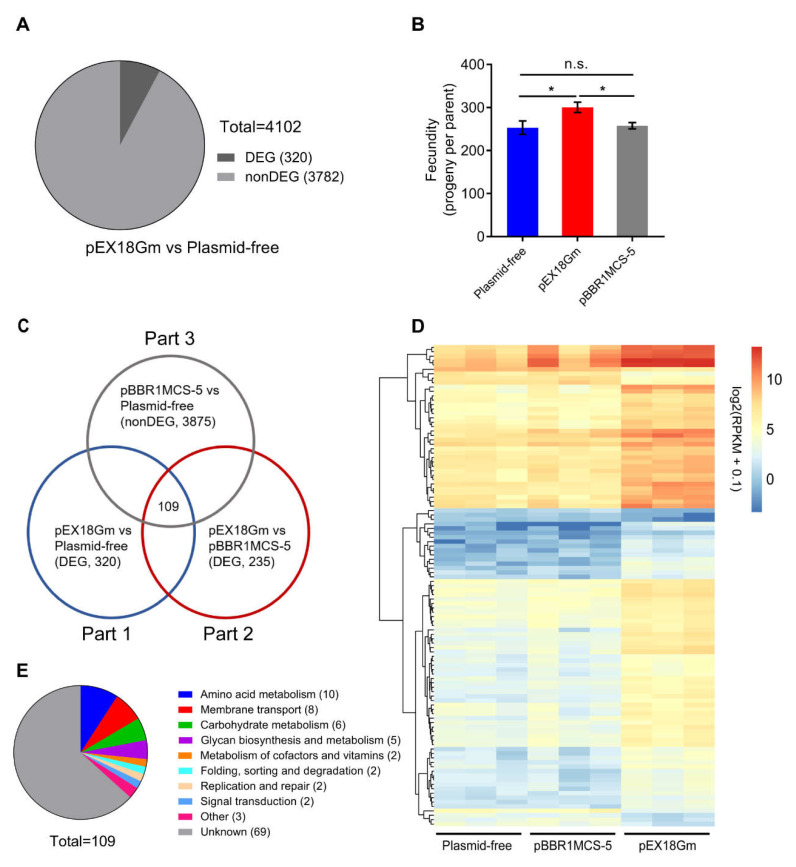
RNA-seq analysis of *E. coli* Strains. (**A**) Pie chart of comparing gene expression between *E. coli* OP50 (plasmid-free) and *E. coli* OP50 harboring plasmid pEX18Gm (pEX18Gm). DEG means significantly differentially expressed genes (*p* < 0.05 and |log2(fold change)| > 1.5); nonDEG means insignificantly differentially expressed genes. (**B**) *C. elegans* fecundity response to feeding on *E. coli* OP50 harboring pEX18Gm and pBBR1MCS-5, respectively. * *p* < 0.05 by Tukey’s multiple comparison test; n.s. indicates not significant. (**C**) Venn diagram of candidate gene screening. Part 1 means significant differential expression genes between *E. coli* OP50 harboring pEX18Gm and plasmid-free *E. coli* OP50. Part 2 means significant differential expression genes between *E. coli* OP50 harboring pEX18Gm and pBBR1MCS-5. Part 3 means insignificant differential expression genes between *E. coli* OP50 harboring pBBR1MCS-5 and plasmid-free *E. coli* OP50. (**D**) Heatmap of 109 candidate gene expression. Expression values (RPKM) were log2 transformed. See Table S1 for details. (**E**) Pie chart about KEGG functional annotation of candidate gene. See Table S1 for details.

To explore the mechanism of pEX18Gm-mediated increases in *C. elegans* fecundity, we performed RNA-seq on *E. coli* OP50 harboring plasmid-free, or harboring pEX18Gm, or pBBR1MCS-5. First, we compared the gene expression of plasmid-free *E. coli* OP50 and *E. coli* OP50 harboring plasmid pEX18Gm and screened 320 significantly different genes among the 4102 genes (Figure 2A). Next, we found that plasmid pEX18Gm can significantly increase the fecundity of *C. elegans* compared with *E. coli* OP50 carrying plasmid-free or pBBR1MCS-5, and plasmid pBBR1MCS-5 cannot increase *C. elegans* fecundity compared with plasmid-free *E. coli* OP50 (Figure 2B). Based on this phenomenon, we further screened out 109 candidate genes from 320 significantly different genes, which may be related to the increased fecundity of *C. elegans* caused by pEX18Gm (Figure 2C). Compared with plasmid-free *E. coli* OP50, the introduction of pEX18Gm resulted in the upregulation of 98 candidate genes and the downregulation of 11 candidate genes (Figure 2D and Table S1). KEGG analysis showed that 41 of these genes are functionally annotated, and that amino acid metabolism accounted for the largest proportion (25%), followed by membrane transport and carbohydrate metabolism (20% and 15%, respectively) (Figure 2E and Table S1).

### 2.3. The Bacterial Methionine Synthase Gene metH Is Necessary for pEX18Gm-Induced Increase in C. elegans Fecundity

**Figure 3:**
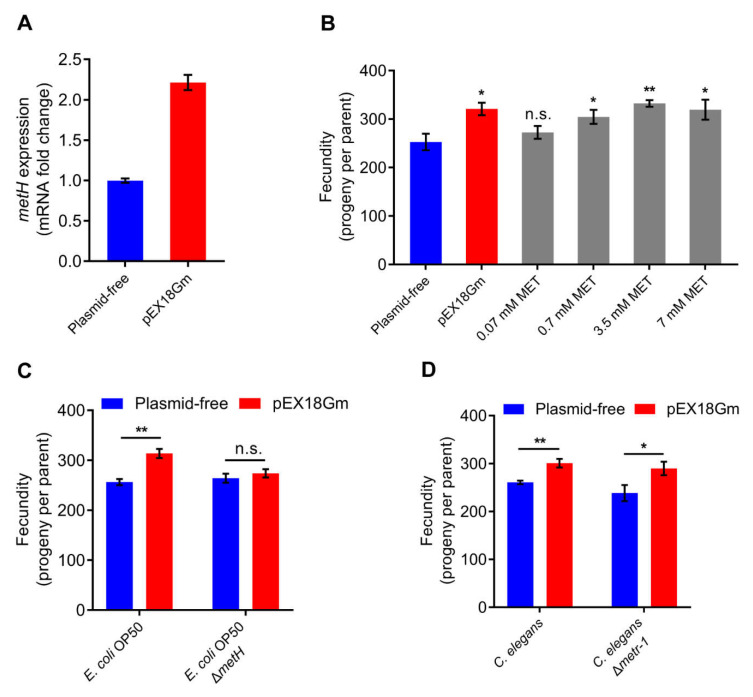
The bacterial methionine synthase *metH* is necessary for pEX18Gm-induced increase in *C. elegans* fecundity. (**A**) mRNA expression of gene *metH* in plasmid-free *E. coli* OP50 and *E. coli* OP50 harboring pEX18Gm. qRT-PCR data, normalized to the *metH* levels in *E. coli* OP50, the house-keeping gene used *gapA*. (**B**) Fecundity of *C. elegans* in *E. coli* OP50 diet on NGM plates supplemented with different concentrations of methionine (MET). (**C**) Effect of pEX18Gm plasmid on the fecundity of *C. elegans* grown on *E. coli* OP50 and mutants *E. coli* OP50 Δ*metH*. *metH* indicates *E. coli* methionine synthase gene. (**D**) Effect of plasmid pEX18Gm on the fecundity of *C. elegans* Δ*metr-1* mutant in *E. coli* OP50 diets. *metr-1* indicates *C. elegans* methionine synthase gene. Error bars indicate ± SEM. * *p* < 0.05; ** *p* < 0.01 by Student’s t test (**A**,**C**,**D**) and Tukey’s multiple comparison test (**B**); n.s. indicates not significant.

To explore which bacterial genes or pathways are responsible for the increased fecundity of *C. elegans* induced by plasmid pEX18Gm, we first reviewed extensive literature and found that only a few substances have been reported to increase animal fecundity on a normal diet, including methionine, resveratrol, polyphenols, and monensin [26,27,28,29,30,31]. From the RNA-seq results, we found that introduction of the pEX18Gm significantly increased the expression of the methionine synthase gene *metH* in *E. coli* OP50 (Figure 2D and Table S1). Then, we performed qRT-PCR to confirm the increased expression of *metH* (Figure 3A). Moreover, we measured the expression of *metH* upstream genes (*metA*, *metB*, *metC*, *malY*, *metR*) in *E. coli*, and found that most upstream genes increased to different degrees in *E. coli* OP50 harboring plasmid pEX18Gm, except for *malY* (Figure S2). Additionally, we fed *C. elegans* with exogenous methionine (MET), and we found that methionine increases the fecundity of *C. elegans* in a range of concentrations (0.7–7 mM) (Figure 3B). Combined with the above results, we propose the hypothesis that pEX18Gm-induced *C. elegans* fecundity improvement is related to methionine synthesis.

Then, we knocked out the gene *metH*, which is responsible for the synthesis of methionine in *E. coli* OP50 [32]. We found that the *E. coli* OP50 harboring plasmid pEX18Gm still increased *C. elegans* fecundity, while the *E. coli* OP50 Δ*metH* mutant harboring the pEX18Gm did not (Figure 3C). This result indicates that the *metH* gene is responsible for the increase in *C. elegans* fecundity. Vitamin B12 is an essential cofactor for MetH, and exogenous Vitamin B12 supplementation can increase the expression of MetH [33]. We found that exogenous Vitamin B12 supplementation significantly increased *C. elegans* fecundity (Figure S3), suggesting that increased MetH expression is sufficient to increase *C. elegans* fecundity. Furthermore, we employed the methionine synthase gene *metr-1* knockout mutant in *C. elegans* [34] and found that the fecundity of *C. elegans* Δ*metr-1* mutant was still increased (Figure 3D), suggesting that the plasmid pEX18Gm does not affect *C. elegans* methionine synthase.

### 2.4. Methionine Is a Metabolite of pEX18Gm-Induced Increase in C. elegans Fecundity

**Figure 4:**
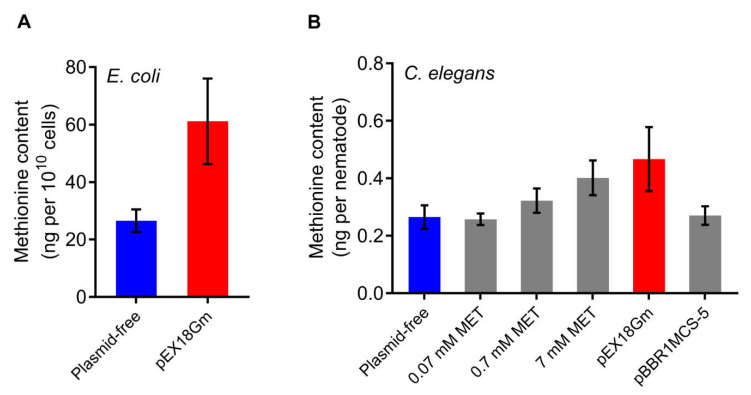
Methionine is a metabolite of pEX18Gm-induced increases in *C. elegans* fecundity. (**A**) Effect of the pEX18Gm plasmid on the methionine content in *E. coli*. (**B**) The methionine content of *C. elegans* in different diets. The treatment is the same as described in Figure 2B and Figure 3B.

To confirm that methionine is the metabolite regulating *C. elegans* fecundity, we measured the content of methionine in *E. coli* and *C. elegans* by high performance liquid chromatography–tandem mass spectrometry (LC-MS/MS). We found that the methionine content in *E. coli* harboring pEX18Gm was 2.3 times higher than that in plasmid-free *E. coli* (Figure 4A). Moreover, the methionine content in *C. elegans* fed with *E. coli* OP50 harboring pEX18Gm was higher than that in *C. elegans* fed with plasmid-free *E. coli* OP50 (Figure 4B). Additionally, we found that methionine content in *C. elegans* was high in the 0.7 MET, 7 MET treatments, but not significantly changed in the 0.07 MET and pBBR1MCS-5 treatments, which is consistent with fecundity changes in different diets (Figure 2B, Figure 3B and Figure 4B). Taken together, these findings indicated that pEX18Gm-induced *C. elegans* fecundity changes are performed by regulating methionine in *E. coli*.

### 2.5. pEX18Gm-Induced Increase in C. elegans Fecundity Is Not Limited to the E. coli OP50 Species

**Figure 5:**
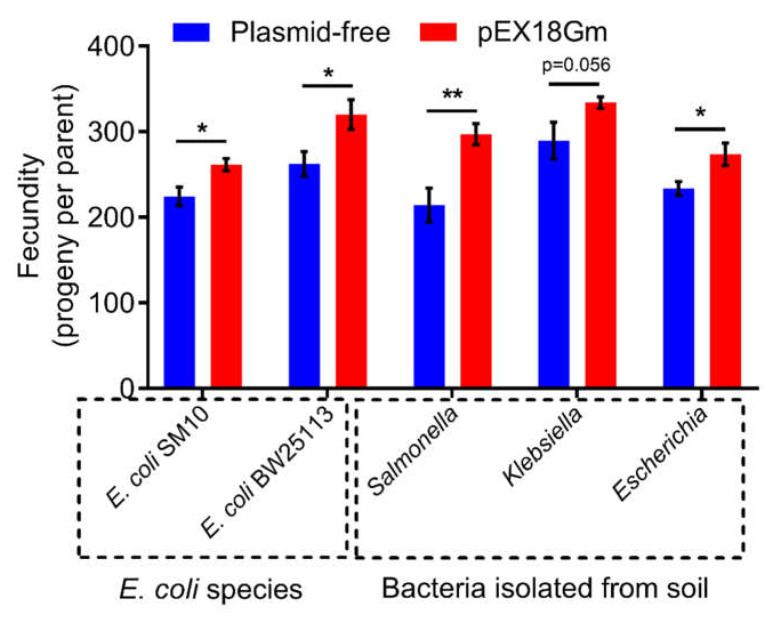
Effects of different bacterial species harboring plasmid pEX18Gm on *C. elegans* fecundity. Error bars indicate ± SEM. * *p* < 0.05; ** *p* < 0.01 by Student’s t test.

Since plasmid has the characteristics of horizontal gene transfer in different bacteria [35], it is worth asking whether the plasmid pEX18Gm-mediated increase in *C. elegans* fecundity is specific to *E. coli* OP50 only. We found that *Salmonella*, *Klebsiella*, *Escherichia* (bacteria isolated from soil), and other *E. coli* strains (*E. coli* SM10, *E. coli* BW25113) harboring pEX18Gm all increased the fecundity of *C. elegans* (Figure 5). These findings indicate that pEX18Gm-induced increases in *C. elegans* can occur in different bacterial species.

## 3. Discussion

In this study, we have established the “plasmid–bacteria–*C. elegans*” model to systematically explore the interaction between plasmids and multi-species. Here, we found that plasmid pEX18Gm increases the methionine content in bacteria by enhancing the expression of the methionine synthase gene *metH*, hence increasing the fecundity of *C. elegans*.

Fecundity is more sensitive than other physiological traits (e.g., lifespan, body size, food consumption and nematode activity) in response to bacteria harboring pEX18Gm (Figure 1). This could be due to the fact that animal reproduction generally responds quickly to changes in diet or environment. For instance, it has been found that *C. elegans*, *Drosophila*, and *rat* will preferentially impair, delay or abolish fecundity in order to survive better under conditions of dietary restriction or nutritional deficiencies [36,37,38,39,40,41]. A cost of reproduction, where lifespan and fecundity are negatively correlated, is of widespread occurrence. However, the trade-off relationship between lifespan and fecundity is not inevitable. We found that *E. coli* OP50 harboring the plasmid pEX18Gm increased *C. elegans* fecundity but did not influence the animal’s lifespan and other life history traits (Figure 1). Therefore, these findings are not in full agreement with the trade-off theory. Gusarov et al. also found a similar phenomenon, the lifespan of *C. elegans* feeding on NOS-deficient *B. subtilis* was reduced by 14.74% compared with worms consuming wild-type bacteria, whereas other physiological parameters, such as fecundity, postembryonic development, and size, were not affected [22]. Some research found that mutations in insulin/IGF signaling (IIS) pathways and dietary restriction (DR) can extend lifespan but do not always reduce fecundity [42,43,44,45]. Therefore, multiple studies provide evidence that does not support the trade-off theory.

From RNA-seq data, we found that the most important impacts of plasmids on host bacterial metabolism are amino acid metabolism and energy metabolism (Figure 2). This result is consistent with other RNA-seq that studies the effect of plasmids on host metabolism [14,15,16,17,18]. Plasmids can affect host physiology by encoding proteins. For example, F and certain R plasmids have been shown to regulate DNA synthesis rates by producing proteins, resulting in increased survival of nitrogen mustard-treated F^-^ cells [9,10]. The plasmid pEX18Gm has two protein-encoding genes, antibiotic resistance gene (*aacC*1) and levansucrase-encoding gene (*sacB*) [46]. First, we employed plasmids pEX18Ap and pEX18Tc, which harbor the same plasmid backbone as pEX18Gm with different antibiotic resistance genes. We found an increase in *C. elegans* fed *E. coli* OP50 harboring any of these three plasmids (Figure S4). Second, we deleted the *sacB* gene from plasmids pEX18Gm, pEX18Ap and pEX18Tc to generate plasmids pEX18GmΔsacB, pEX18ApΔsacB and pEX18TcΔsacB. We found that the fecundity of *C. elegans* was increased when feeding *E. coli* OP50 harboring any of these plasmids (Figure S5). These findings suggest that the plasmid pEX18Gm did not regulate the *C. elegans* fecundity by encoding the protein. Moreover, plasmids can regulate host physiology by encoding RNA. For instance, the pPCP1 plasmid encodes a high-abundance *HmsA* sRNA, which can promote biofilm formation and c-di-GMP synthesis in *Yersinia pestis* [47]. Thus far, no studies have shown that plasmid pEX18Gm possesses RNA-encoding elements. Further studies are needed on the mechanism by which plasmid pEX18Gm regulates bacterial methionine metabolism.

Grandison et al. tested ten essential and ten non-essential amino acids and found that methionine alone is sufficient to increase fecundity of *Drosophila*, while other amino acids have no effect on reproduction [29]. Additionally, supplementing methionine in the diet to increase fecundity has been widely used in animal breeding such as cows, quails, rams [48,49,50]. We found that methionine is the metabolite of pEX18Gm-induced changes in *C. elegans* fecundity. However, the mechanism of how methionine regulates reproduction is still unclear. Grandison et al. found that dietary methionine supplementation is unable to promote fecundity in IIS mutants, suggesting that methionine effect in promoting fecundity also relies on IIS pathways [29]. In the green pea aphid, the accumulated methionine enhanced the target of the rapamycin (TOR) signaling pathway, which consequently increased fecundity by promoting vitellogenin synthesis [51]. Therefore, the nematode IIS and TOR pathways are the possible pathways for the plasmid pEX18Gm to increase the fecundity of *C. elegans*.

Our study shows that plasmid pEX18Gm increases the fecundity of *C. elegans* mediated by bacteria, which is not only detected with *E. coli* but also with other bacteria (Figure 5). This line of evidence raises the intriguing possibility that natural plasmids carrying similar functions as pEX18Gm can probably influence the soil food web including bacterial and nematode communities. Our study using a “plasmid–bacteria–*C. elegans*” model also provides a new insight into plasmid–host interactions because we show that plasmids such as pEX18Gm have clear impact on certain physiological traits of the host bacteria as well as their forager.

## 4. Materials and Methods

### 4.1. Plasmids and Chemicals

Plasmids pEX18Gm (GenBank: AF047518.1) [46], pEX18Ap (GenBank: AF004910.1) [46], pEX18Tc (GenBank: AF047519.1 [46]), pBBR1MCS-2 (GenBank: U23751.1) [52], pBBR1MCS-3 (GenBank: U25059.1) [52], pBBR1MCS-5 (GenBank: U25061.1) [52] and pACYC184 (GenBank: X06403.1) [53] were kind gifts from Pro. Jun Wu (Soil Ecology Lab, Nanjing Agricultural University, China). Plasmids pSC101_TIMER [54] and R6K_BOX were purchased from Miaoling Plasmid Platform (Wuhan, China). The *E. coli* gene knockout plasmids pKD46, pKD3, and pCP20 were kind gifts from Pro. Xin Yan (Key Laboratory of Agricultural Environmental Microbiology, Nanjing Agricultural University, China).

Methionine (MET), Ampicillin (Ap) and chloramphenicol (Cm) were purchased from Sigma Aldrich (St. Louis, MO, USA). Gentamicin (Gm) and Tetracycline (Tc) were purchased from Macklin (Shanghai, China). Kanamycin (Km) was purchased from Aladdin (Shanghai, China). Antibiotics were used in the following concentrations: Ap: 100 μg/mL; Cm: 50 μg/mL; Gm: 50 μg/mL; Tc: 5 μg/mL; Km: 50 μg/mL. Antibiotic-gradient plates were made applying the protocol of Szybalski and Bryson [55].

### 4.2. Strains and Growth Conditions

Bacterial strains in this study are listed in Table 2. Unless otherwise noted, Bacteria were grown in liquid Luria–Bertani (LB) medium (10 g tryptone, 5 g yeast extract, 5 g NaCl, H_2_O to 1 L, autoclaved), or on LB plates containing 1.5% agar. All *E. coli* strains were cultivated at 37 °C. The bacteria *Salmonella*, *Klebsiella*, and *Escherichia* were cultivated at 30 °C.

*C. elegans* strains in this study are listed in Table 2. The nematodes were maintained at 20 °C on nematode growth medium (NGM) (3 g NaCl, 2.5 g peptone, 17 g agar and 975 mL H_2_O; the medium was autoclaved, cooled to 55 °C, and supplied with 25 mL 1M KPO_4_ buffer (pH 6.0), 1 mL 1M CaCl_2_, 1 mL 1M MgSO_4_ and 1 mL 5 mg/mL cholesterol in ethanol that had been filtered through a 0.22 μm filter) [21]. Unless otherwise noted, *C. elegans* indicates *C. elegans* N2 (Bristol) as the wild-type strain. The mutant *C. elegans* Δ*metr*-1 (*ok 521*) was backcrossed 3 times against N2 prior to assays.

### 4.3. Competent Cells Preparation and Plasmid Introduction

Overnight cultured bacteria were diluted 1:100 in LB and on a Tecan Spark plate reader with the absorbance (OD_600_ nm) recorded every 30 min. When the bacterial OD_600_ reached about 0.5, it was placed on ice and pre-cooled for 30 min. Then, the bacteria were concentrated by centrifugation at 4 °C, 12,000 rpm for 5 min, and washed 3 times with pre-cooled sterile water. The bacteria pellets were resuspended with a small amount of 0.1 M GaCl_2_. The plasmids were mixed with the resuspend bacteria, and BIO-RED MicroPulser (Hercules, CA, USA) was used for electroporation transformation. The cells were recovered in 1 mL of LB and shaken for 1–2 h at 30 or 37 °C (depending on the specific case). Finally, 10–100% of the recovery culture was plated on LB agar plates harboring the appropriate antibiotic. The selected positive clones will be further confirmed by plasmid extraction and sequencing to ensure that the plasmids are introduced into bacteria.

### 4.4. Phenotypic Analysis of C. elegans Life History Traits

#### 4.4.1. Fecundity

Animals were grown on a relevant diet, and eggs were collected by bleaching, washed three times in M9 buffer (5 g NaCl, 3 g KH_2_PO_4_, 6 g Na_2_HPO_4_, 1 mL 1 M MgSO_4_, H_2_O to 1 L, autoclaved), and allowed to hatch in M9 buffer for 18 h. Following synchronization [21], animals were transferred to NGM plates and incubated at 20 °C. Ten individual L4 animals (L4 is a stage of pre-maturity after which *C. elegans* will spawn) were placed on NGM plates seeded with different diets. Animals were transferred daily, and number of offspring on the plates was counted. Incomplete offspring counts due to escaping or dying mothers were left out of the data analyses. Fecundity was determined by adding offspring produced across all days. The *C. elegans* fecundity assays in each panel are independent; therefore, the same treatments have different values in different panels.

#### 4.4.2. Lifespan

One hundred L4 animals of each treatment were divided into 10 replicates and transferred on NGM plates seeded with relevant diet. The following day, the animals were transferred to NGM plates seeded with the appropriate bacteria. Every day, animals were checked for pharyngeal pumping. If pumping was not observed, animals were lightly prodded with a platinum wire. If animals did not respond, they were considered dead and were scored and removed.

#### 4.4.3. Body Size

Approximately 25 synchronized L1 animals were placed on NGM plates seeded with different diets and were allowed to develop for 48 h at 20 °C. Animals were then washed with M9 buffer and transferred to fresh 96-well plates. Bright-field images of animals in each well were collected using an Invitrogen EVOS FL imaging system, and body size was determined by measuring the surface area of the animals using ImageJ [56].

#### 4.4.4. Pharyngeal Pump Rates

Ten L4 animals were randomly selected from NGM plates which seeded with different diets. Pharyngeal contractions in 30 s periods were counted using a Sony camera attached to a Zeiss microscope. The pumping rates per minute were calculated.

#### 4.4.5. Body Bends

L4 animals growing on different diets were washed into plates containing M9 buffer. After calming down for 30 min, 10 animals were randomly selected under the microscope to record the number of body swings within 1 min.

### 4.5. Bacterial Growth Rate

Liquid bacterial growth was performed in LB glass tube containing the respective bacterial strain (previously grown overnight in LB and diluted 1:1000). The absorbance (OD_600_ nm) was measured every 1 h for incubation period with shaking at 37 °C, 200 rpm using a Tecan Spark plate reader. When the OD_600_ value reached a plateau, the measurement was terminated. Data analysis was performed on 3 replicate trials for each condition. Values for graphs were taken from OD_600_ values at 10 h of growth.

### 4.6. RNA-seq and Analysis

Bacteria cultured overnight in LB medium were collected by centrifugation, washed 3 times with sterile water and flash frozen in liquid nitrogen. Total RNA was extracted using TRIzol (Invitrogen, San Diego, CA, USA), followed by DNase I (TaKaRa, Dalian, China) treatment and purified using the Ribo-Zero Magnetic kit (TaKaRa, Dalian, China). RNA quality was verified by agarose gel electrophoresis and assessed by Agilent 2100 Bioanalyzer and NanoDrop for quality control. Three biological replicates were sequenced on an Illumina HiSeq 4000 platform (Shanghai Biozeron Biological Technology Co. Ltd., Shanghai, China), and 150 base pair paired-end reads were generated. Raw reads obtained by sequencing were cleaned by filtering with Trimmomatic software. After quality control with NGS QC Toolkit, the clean reads were mapped to the reference genome using Bowtie 2. The maximum number of allowed mismatched bases per read was set at 0; the other parameters were set as the software defaults. The expression levels of genes were measured by reads per kilobase of transcript per million reads mapped (RPKM). HTSeq software was used to analyze the gene expression levels of different samples. Differentially expressed genes of E. coli under different treatments were identified (*p* < 0.05 and |log2(fold change)| > 1.5). The selected candidate genes were subjected to Kyoto Encyclopedia of Genes and Genomes (KEGG) for gene function annotation.

The RNA-sequencing data files were deposited in the NCBI Sequence Read Archive (SRA) under the accession number PRJNA746633.

### 4.7. qRT-PCR

Overnight cultured bacteria strains in LB were washed twice in sterile water, pelleted by centrifugation, and frozen at −80 °C in TRIzol (Invitrogen, San Diego, CA, USA). RNA was collected using TRIzol extraction by Dnase I treatment. Then, 1 μg total DNA-free RNA was reverse transcribed using the Primerscript RT reagent kit (TaKaRa, Dalian, China). Depending on the target of interest, cDNA was amplified with the corresponding primers of *metH*. qPCR was performed using Eppendorf Mastercycler RealPlex2 and TB green premix ex taq (TaKaRa, Dalian, China) as suggested by the manufacturers. PCR conditions consisted of one cycle at 95 °C for 15 min; 40 cycles at 94 °C for 20 s, 56 °C for 20 s, 72 °C for 50 s; and a final cycle at 94 °C for 15 s. Relative mRNA expression was calculated by the ΔΔCT method and the house-keeping gene used *gapA*. qRT-PCR primers used in the study are listed in Table S2.

### 4.8. E. coli Gene Knockout

Unmarked gene knockout in *E. coli* OP50 was performed according to a previously reported method [57]. To confirm the correctness of the primer design, the upstream homologous arm (UHA) and the downstream homologous arm (DHA) were amplified from *E. coli* OP50 genomic DNA using the primer *metH*-UHA and *metH*-DHA, respectively. The chloramphenicol resistant cassette (CRC) was amplified from pKD3 using the homologous recombination primer *metH*-CRC. The PCR product was directly introduced to pKD46-containing *E. coli* OP50 competent cells. The transformants were selected on LB plates containing chloramphenicol and confirmed by DNA sequencing with primer *metH*-con. The pCP20 plasmid was introduced into the transformants and incubated at 30 °C and 42 °C for 8 h respectively. Transformants that can grow on non-resistant LB plates but cannot survive on chloramphenicol LB plates are Δ*metH* mutants. The mutant was further confirmed by PCR and DNA sequencing. The primers used in the study are listed in Table S2.

### 4.9. Methionine and Vitamin B12 Supplement Assay

A 350 mM stock solution of methionine was made in ddH_2_O and filter sterilized. The stock solution was diluted to final concentration (0.07, 0.7, 3.5, 7 mM) in NGM agar prior to plate pouring. A 5 mM stock solution of Vitamin B12 was made in ddH_2_O and filter sterilized. The stock solution was diluted to final concentration (6.4, 64, 640 nM) in NGM agar prior to plate pouring. Synchronized L1 *C. elegans* were inoculated into the relevant NGM plate and grown on the *E. coli* OP50 diet. *C. elegans* fecundity was measured.

### 4.10. Methionine Determination in Bacteria and C. elegans by LC-MS/MS

#### 4.10.1. Bacteria Sample Preparation

The bacteria were cultured in liquid mineral medium (10 g glucose, 5 g glycerin, 5 g NaCl, 2 g KH_2_PO_4_, 6 g K_2_HPO_4_, 5 g (NH_4_)_2_SO_4_, 0.2 g MgSO_4_ with 1 L H_2_O) at 37 °C with shaking at 200 rmp. Overnight cultured bacteria strains were centrifuged at 20 °C, 8000 rpm for 10 min and then resuspended and washed four times with sterile water. The sample was divided into two parts: one part was used for colony forming unit counts to calculate the number of bacteria, and the other part was sonicated for 5 min using a hand-held sonicator at 40% amplitude on ice. Suspensions were filtered with a 0.45 μm filter and directly analyzed by LC-MS/MS.

#### 4.10.2. *C. elegans* Sample Preparation

L4 animals growing on different diets were collected. Animals were washed six times using sterile water to remove bacteria. The number of animals was counted and then sonicated for 30 s using a hand-held sonicator at 40% amplitude on ice. Suspensions were filtered with a 0.45 μm filter and directly analyzed by LC-MS/MS.

#### 4.10.3. LC-MS/MS Analysis

To quantify the content of methionine, methionine standards with concentrations of 1, 0.75, 0.5, 0.25, 0.1, and 0.05 ug/mL were prepared. The samples and methionine standards were analyzed using an Agilent Technologies 1200 Series RRLC system coupled with an Agilent 6410B triple quadrupole mass spectrometer (Agilent Technologies Inc., Santa Clara, CA, USA) equipped with an electrospray source (ESI, Agilent Technologies Inc., Santa Clara, CA, USA). An Agilent ZORBAX Plus C18 reverse phase column (2.1 × 150 mm; 3.5 μm) (Agilent Technologies Inc., Santa Clara, CA, USA) with mobile phases A (acetonitrile) and B (0.1% formic acid in water) (2:8, *v*:*v*) was used, with the column temperature set to 30 °C and a flow rate of 0.2 mL/min. The total run time was 8 min, and the injection volume was 5 μL. The positive ionization mode was used at a spray voltage of 4000 V. The nebulizer gas (N_2_) pressure was set to 15 psi with a flow rate of 6 L/min. The heated capillary temperature was 300 °C.

### 4.11. Statistical Analysis

Statistical analysis was performed with GraphPad Prism 7 software (GraphPad Softioare Inc., San Diego, CA, USA). Statistical significance for experimental data was determined using Student’s *t*-test or Tukey’s multiple comparison test, and *p* values less than 0.05 were taken to indicate statistical significance.

## Figures and Tables

**Table 1:** Plasmid information used in this study.

Name	Size	Antibiotic Resistance	Replicon	Copy Number Level
pEX18Gm	5831 bp	Gm	pMB1	high
pBBR1MCS-2	5144 bp	Km	P15A	high
pBBR1MCS-3	5228 bp	Tc	P15A	high
pBBR1MCS-5	4768 bp	Gm	P15A	high
pACYC184	4245 bp	Cm, Tc	P15A	low
pSC101_TIMER	4946 bp	Km	pSC101	low
R6K_BOX	1801 bp	Km	R6K	high

Abbreviations: Gm, gentamicin; Km, kanamycin; Tc, tetracycline; Cm, chloramphenicol.

**Table 2:** Strains used in this study.

	Strain	Source	Identifier
Bacterial strain	*E. coli* OP50	*Caenorhabditis* Genetics Center	N/A
	*E. coli* OP50 Δ*metH*	This work	N/A
	*E. coli* DH5α	Vazyme	Cat#: C502
	*E. coli* SM10	Miaolingbio	Cat#: S0049
	*E. coli* BW25113	Dharmacon	Cat#: OEC4988
	*Salmonella*	This work	N/A
	*Klebsiella*	This work	N/A
	*Escherichia*	This work	N/A
Nematode strain	*C. elegans* N2	*Caenorhabditis* Genetics Center	N/A
	*C. elegans* Δ*metr*-1	*C. elegans* Gene Knock-out Consortium	N/A

## Data Availability

Some or all data during the study are available from the corresponding author by request.

## Supplementary Materials

The following supporting information can be downloaded at: https://www.mdpi.com/article/10.3390/ijms23095003/s1.
