# Supplementary material for: The Plasmid pEX18Gm Indirectly Increases Caenorhabditis elegans Fecundity by Accelerating Bacterial Methionine Synthesis
Source: Int J Mol Sci. 2022 Apr 30;23(9):5003. doi: 10.3390/ijms23095003 (PMC9102816; doi:10.3390/ijms23095003)
Supplement: Supplementary file 1 [file ijms-23-05003-s001.zip › Figure S2. mRNA expression of methionine synthesis related genes in plasmid-free E. coli OP50 and E. coli OP50 harboring pEX18Gm.pdf]

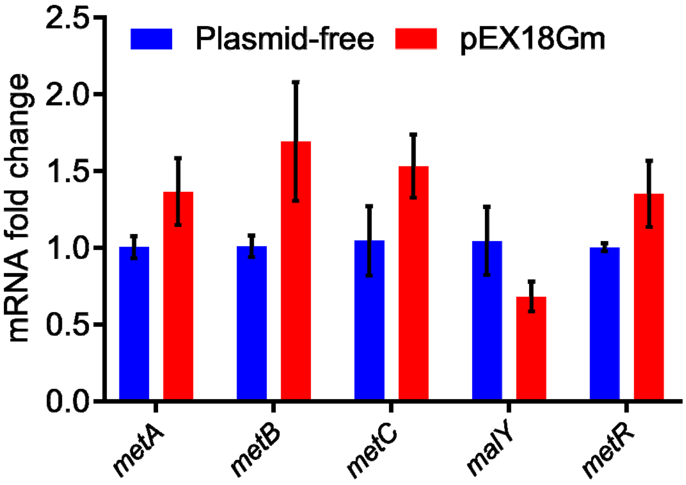

Figure S2. mRNA expression of methionine synthesis related genes in plasmid-free *E. coli* OP50 and *E. coli* OP50 harboring pEX18Gm. qRT-PCR data, normalized to the gene levels in *E. coli* OP50, the house-keeping gene used *gapA*.
