# Supplementary material for: The Plasmid pEX18Gm Indirectly Increases Caenorhabditis elegans Fecundity by Accelerating Bacterial Methionine Synthesis
Source: Int J Mol Sci. 2022 Apr 30;23(9):5003. doi: 10.3390/ijms23095003 (PMC9102816; doi:10.3390/ijms23095003)
Supplement: Supplementary file 1 [file ijms-23-05003-s001.zip › Figure S4. C. elegans fecundity response to feeding on E. coli OP50 harboring pEX18Gm, pEX18Ap and pEX18Tc, respectively.pdf]

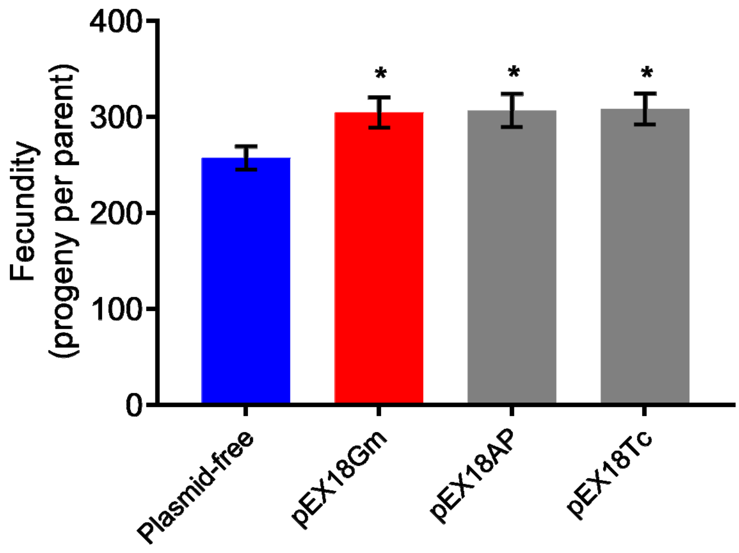

Figure S4. *C. elegans* fecundity response to feeding on *E. coli* OP50 harboring pEX18Gm, pEX18Ap and pEX18Tc, respectively. *$p < 0.05$ by Tukey multiple comparison test.
