# Supplementary material for: The Plasmid pEX18Gm Indirectly Increases Caenorhabditis elegans Fecundity by Accelerating Bacterial Methionine Synthesis
Source: Int J Mol Sci. 2022 Apr 30;23(9):5003. doi: 10.3390/ijms23095003 (PMC9102816; doi:10.3390/ijms23095003)
Supplement: Supplementary file 1 [file ijms-23-05003-s001.zip › Figure S5. C. elegans fecundity response to feeding on E. coli OP50 harboring pEX18GmañsacB, pEX18ApañsacB and pEX18TcañsacB, respectively.pdf]

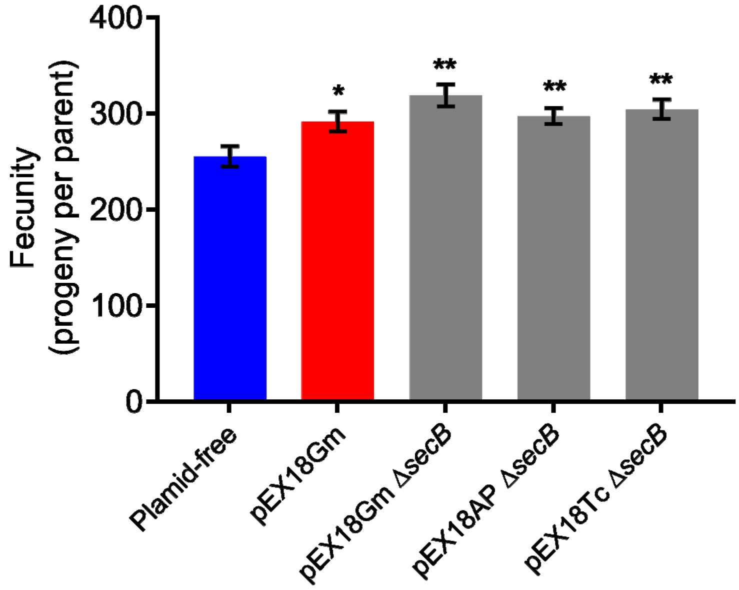

Figure S5. *C. elegans* fecundity response to feeding on *E. coli* OP50 harboring pEX18GmΔ*sacB*, pEX18ApΔ*sacB* and pEX18TcΔ*sacB*, respectively. Error bars indicate ± SEM. *$p < 0.05$; **$p < 0.01$ by Tukey multiple comparison test.
