# Supplementary figures and images for: The Plasmid pEX18Gm Indirectly Increases Caenorhabditis elegans Fecundity by Accelerating Bacterial Methionine Synthesis
Source: Int J Mol Sci. 2022 Apr 30;23(9):5003. doi: 10.3390/ijms23095003 (PMC9102816; doi:10.3390/ijms23095003)

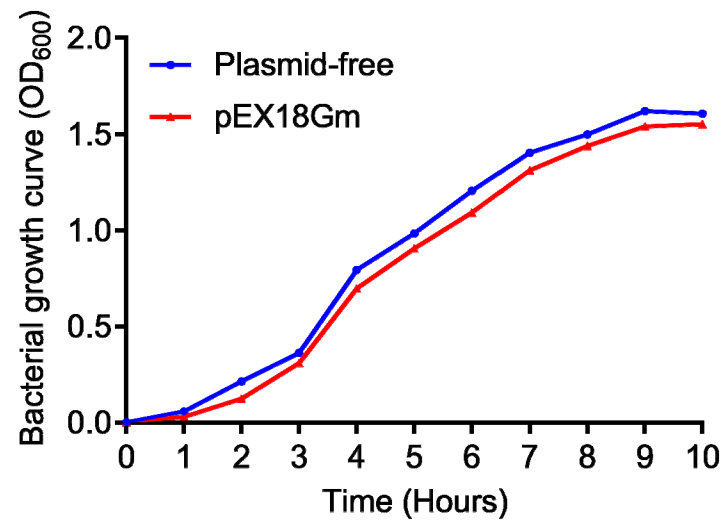

**Figure S1.** The response of *E. coli* OP50 growth rate to plasmid pEX18Gm in liquid LB medium.

Supplement: Supplementary file 1 [file ijms-23-05003-s001.zip › Figure S1. The response of E. coli OP50 growth rate to plasmid pEX18Gm in liquid LB medium.pdf]
